# Intercellular Adhesive Structures Between Stellate Cells – An Analysis in Cultured Human Hepatic Stellate Cells

**DOI:** 10.1186/1476-5926-2-S1-S13

**Published:** 2004-01-14

**Authors:** Katsuyuki Imai, Mitsuru Sato, Takeya Sato, Naosuke Kojima, Mitsutaka Miura, Nobuyo Higashi, Da-Ren Wang, Shinsuke Suzuki, Haruki Senoo

**Affiliations:** 1 Department of Anatomy, Akita University School of Medicine, Akita 010-8543, Japan

## Abstract

To investigate whether or not hepatic stellate cells can form intercellular junctions with each other, we cultured human stellate cells (LI90) on different kinds of substrata. Intercellular junctions were detected between these cultured stellate cells by transmission electron microscopy (TEM). The molecular components of the intercellular adhesive structures were identified by immunofluorescence microscopy. Immunofluorescence for cadherin and catenins was detected at the adhesion sites between the cultured stellate cells. Thus, the intercellular junctions were indicated to be adherens junctions at the molecular level. The junctions developed in the cultured stellate cells irrespective of the type of substratum. These data suggest that the junctional formation between the stellate cells occurs *in vivo *as well as *in vitro*.

## Introduction

In the normal liver, hepatic stellate cells are located between sinusoidal endothelial cells and hepatic parenchymal cells. The stellate cells and their processes adhere to the endothelial cells on one hand and the other hand contact with the parenchymal cells [1]. However, the presence of intercellular junctions between stellate cells has not yet been fully established. The processes of stellate cells coexist with the three-dimensional extracellular matrix (ECM) within the space [2,3]. The three-dimensional ECM components and the long numerous processes of stellate cells make a complicated structure. Hence, it is difficult to elucidate cell-cell junctions between hepatic stellate cells *in vivo*. To investigate whether or not hepatic stellate cells can form intercellular junctions with each other, we cultured stellate cells, and then morphologically examined the structure of the cultured cells with special reference to intercellular junctions.

## Methods

For morphological examinations by electron microscopy, we cultured LI90 cells on the different types of substrata, namely, non-coated polystyrene culture dishes, type I collagen gel, or Matrigel. The structures of cultured LI90 cells were examined with special reference to intercellular adhesive structures in the cultured cells by TEM. For immunofluorescence microscopy, we seeded LI90 cells into glass-bottom dishes. The molecular components of the intercellular adhesive structures formed by the cultured cells were identified by indirect immunofluorescence microscopy for pan-cadherin, alpha- and beta-catenins. Immunostained cells were examined with a confocal laser-scanning microscope (LSM).

## Results

**Figure 1:**
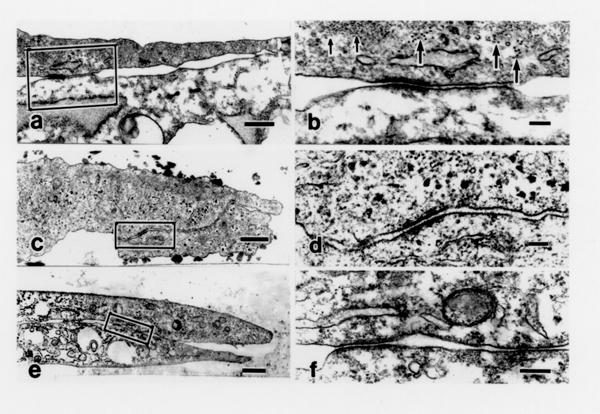
Transmission electron micrographs of hepatic stellate cells cultured in non-coated polystyrene culture dishes (panel **a**, **b**), on type I collagen gel (panel **c**, **d**), or on Matrigel (panel **e**, **f**). Panel **a**: close cell-cell contacts are detected between the stellate cells in non-coated polystyrene dishes. Bar = 500 nm. Panel **b**: enlarged image of the part of panel "**a**" indicated by the rectangle. Thin filaments and intermediate filaments are indicated by small- and large-sized arrows, respectively. Bar = 100 nm. Panel **c**: transmission electron micrograph of the cultured stellate cells on type I collagen gel. Bar = 500 nm. Panel **d**: enlarged image of the part of panel "**c**" marked by the rectangle. Bar = 100 nm. Panel **e**: transmission electron micrograph of the stellate cells on Matrigel. Bar = 1 –m. Panel **f**: enlarged image of the part of panel "**e**" covered by the rectangle. Bar = 200 nm. The cultured cells were examined with a TEM as described under Materials and Methods.

Morphology of the site of adhesion between the cultured stellate cells was revealed by TEM observation (Fig. 1a,1b,1c,1d,1e,1f). Stellate cells cultured in non-coated polystyrene culture dishes (Fig. 1a,1b or on type I collagen gel (Fig. 1c,1d) formed intercellular junctions. At the sites of intercellular junctions between the cells, plasma membranes of the cells were arranged in parallel and characterized by electron-dense cytoplasmic plaques. The widths of the intercellular space were less than 20 nm. Microfilaments, having an average diameter of 6 nm (small-sized arrows in Fig. 1b), and intermediate filaments, with an average diameter of 10 nm (large-sized arrows in Fig. 1b), were observed near the junctions. The same characteristic intercellular junctions were also observed between the stellate cells cultured on Matrigel (Fig. 1e,1f). Intercellular junctions developed in cultured stellate cells irrespective of the sort of substratum.

**Figure 2:**
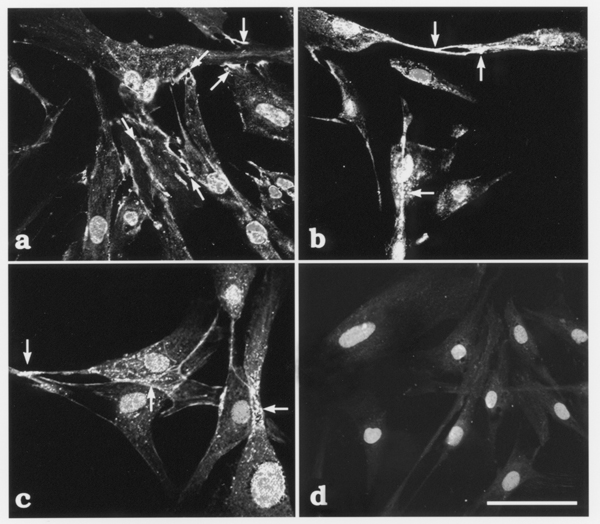
Immunofluorescence localization of pan-cadherin (panel **a**), alpha-catenin (panel **b**), and beta-catenin (panel **c**) in the LI90 cells cultured on glass-cover-slip in the bottom of culture dishes. Nuclei of the cultured cells were counterstained with propidium iodide (panel **a**–**d**). The cultured cells were examined with an LSM as described under Materials and Methods. Strong immunofluorescence for pan-cadherin was observed at the sites of intercellular contact (arrows in panel **a**) or peripheral portion of the cells. Immunofluorescence for alpha-catenin was observed in the cell processes (arrows in panel **b**). Immunofluorescence for beta-catenin was clearly observed as discontinuous dot-like labeling where the cultured cells overlapped one another (arrows in panel **c**). As a control, some cultured cells grown on glass-bottom dishes were prepared with omission of the primary antibodies from the staining procedure. In these control specimens, fluorescence of Alexa Fluor 488 was completely negative in the cultured cells, although fluorescence of propidium iodide was clearly observed in the nuclei of the cells (panel **d**). Bar = 100 –m.

Immunofluorescence for pan-cadherin (Fig. 2a), alpha-catenin (Fig. 2b), and beta-catenin (Fig. 2c) was observed at the sites of intercellular contact (arrows in Fig. 2a,2b,2c) between cultured stellate cells. In the control specimens, fluorescence of Alexa Fluor 488 was completely negative in the cultured cells, although the fluorescence of propidium iodide was clearly observed in their nuclei (Fig. 2d).

## Discussion

Cultured LI90 cells formed intercellular adhesive structures with each other. At the sites of intercellular junctions, plasma membranes of the cells were arranged in parallel and characterized by electron-dense cytoplasmic plaques. The adhesive structures were morphologically identified as adherens junctions at the fine structural level. As cadherin family, alpha- and beta-catenins are molecular components of adherens junctions [4], the intercellular adhesive structures formed by the cultured LI90 cells were presumed to be adherens junctions. Stellate cells coexist with many kinds of extracellular matrix components within the normal perisinusoidal space [1-3]. The junctions developed in the cultured stellate cells irrespective of the type of culture substratum. These data suggest that the junctional formation between stellate cells occurs *in vivo *as well as *in vitro*. Thus, the hepatic stellate cells may participate in the structural organization of the cells in liver lobules through the formation of intercellular junctions between themselves.
